# Combating Kidney Fibrosis

**DOI:** 10.1155/2014/679154

**Published:** 2014-09-07

**Authors:** Keizo Kanasaki, Akito Maeshima, Gangadhar Taduri, Ignacio Revuelta

**Affiliations:** ^1^Department of Diabetology & Endocrinology, Kanazawa Medical University, Kahoku, Ishikawa 920-0293, Japan; ^2^Department of Medicine and Clinical Science, Gunma University Graduate School of Medicine, Maebashi, Gunma 371-8511, Japan; ^3^Department of Nephrology, Nizams Institute of Medical Sciences, Hyderabad, Andhra Pradesh 500034, India; ^4^Renal Transplant Unit, Department of Nephrology and Renal Transplant, Hospital Clinic, IDIBAPS, University of Barcelona, 08036 Barcelona, Spain

An estimated 10% of the world population has some form of kidney disease. Kidney fibrosis is the final common pathway of progressive kidney diseases, resulting in subsequent massive destruction of normal kidney structure and diminishing the function. Currently approved therapies are neither pathway nor cell specific in nature, due to which these therapies became ineffective in reducing the fibrosis and are associated with side effects. The understanding of the pathways and cells that are involved in the fibrosis will guide the future therapies to combat the kidney fibrosis.

In this special issue of the BioMed Research International, we have designed to invite original as well as review articles regarding the pathophysiologic clue to combat kidney fibrosis in various diseases.

In a research article Takako Nagai et al. (Kanazawa Medical University, Japan) focused on the endogenous antifibrotic peptide, N-acetyl-seryl-aspartyl-lysyl-proline (Ac-SDKP), one of the substrates of angiotensin converting enzyme (ACE), and found that AcSDKP suppressed kidney fibrosis in diabetes or even restored normal kidney structure from damaged kidney associated with the inhibition of endothelial mesenchymal transition and the induction of fibroblast growth factor receptor-microRNA let-7 axis.

There are two other research articles in the topic of diabetic nephropathy from the research team led by Yong Xu (Luzhou Medical College, China). Huang et al. found that proteasome inhibitor MG132 inhibited profibrotic cytokine transforming growth factor (TGF)-*β* signaling via endonuclear transcription corepressor SnoN degradation and ameliorated diabetic nephropathy. Another paper from Yong's research team by Zhou et al. found that high glucose in mesangial cells induced sumoylation (by SUMO 2/3) of smad4, the co-smad essential for TGF-*β*-induced signal transduction, and such sumoylation would be important for enhanced TGF-*β* signal transduction in the cells exposed to high glucose condition and diabetic kidney.

Focusing on TGF-*β*-induced profibrotic signaling pathway on the ligand-receptor complex level, there are two exciting papers included in the special issue. Maeshima et al. (Gunma University Graduate School of Medicine, Japan) found that activin A, a member of TGF-*β* superfamily, exhibited profibrotic action in unilateral ureteral obstruction (UUO) model. They showed that UUO kidney displayed significant induction of activin A in the interstitial *α*SMA-positive fibroblasts and follistatin, an activin antagonist, significantly reduced the fibrotic area in the UUO kidney, suggesting the essential role of activin A signaling in the development of interstitial fibrosis in this model, and its antagonist could be a novel approach for the prevention of kidney fibrosis.

Another paper by Rodrigues-Diez et al. (Universidad Autonoma Madrid, Spain) focused on gremlin, a well-known bone morphogenetic proteins (BMPs) antagonist. They found that gremlin induced early activation of smad2/3 signal transduction via TGF-*β* independent manner in human tubular epithelial cells and long-term exposure of gremlin induced epithelial mesenchymal transition (EMT). Such long-term exposure of gremlin-induced EMT was diminished by TGF-*β* neutralizing antibody, suggesting that, different from early effects, TGF-*β* induction was involved in the long-term exposure.

UUO is the model that researchers frequently used for kidney fibrosis research. Therefore the biology of UUO would provide meaningful information for future kidney fibrosis research. In regard to this, Rodrigues-Pena et al. (Universidad de Salamanca, Spain) focused on early fibrotic changes of UUO and found that activation of RAS pathway would be the clue to inhibit fibrotic changes of UUO by confirming the potent inhibitor of this pathway utilizing angiotensin II, losartan, atorvastatin, and farnesyl transferase inhibitors.

Banon-Maneus et al. (Ludwig-Maximilians-University, Germany, and laboratorio Experimental de Nefrologia Transplant, Spain) focused on Wnt/*β*-catenin pathway for the therapeutic target to combat kidney fibrosis. It is well known the significance of Wnt/*β*-catenin pathway in several human diseases and abnormal activation of Wnt/*β*-catenin pathway is associated with progressive damage and organ failures. In their paper, authors confirmed that activation of Wnt/*β*-catenin pathway was involved in 5/6 renal mass reduction model (RMR) and suggested that RMR is nice animal model for aberrant activation of Wnt/*β*-catenin pathway to perform experimental therapy by various molecules.

Finally, the special issue included exceptional 5 review articles. Maeshima et al. (Gunma University Graduate School of Medicine, Japan) summarized recent advance in regenerative medicine for kidney. Renotropic factors, renal stem/progenitor cells, and stem cell therapies are examined in this review, and the authors discussed the issues to be solved to realize regenerative therapy for kidney diseases in humans. Kim et al. (Pusan National University School of Medicine, Korea) well described the role of uric acid in kidney fibrosis and also suggested future direction of interventional research to proof causal relationship between uric acid and kidney fibrosis development. Kume et al. (Shiga University of Medical Science, Shiga, Japan) discussed the roles of nutrient sensing pathway, such as mTORC1, AMPK, and sirt1, in the development of diabetic nephropathy. They provided a excellent summary of recent advances in this field and made very informative tables for other researchers who investigate the signaling pathway associated with nutritional responses. Torres et al. (Autonomous University of Barcelona, Spain) wrote very unique, informative, and educative review about the interaction between inflammation and fibrosis in transplanted kidney. In spite of previous hypothesis, early subclinical inflammation may trigger the chronic sequence of kidney fibrosis, resulting in kidney graft failure. The authors reviewed the different immunosuppressive protocols used in kidney transplantation, focusing in minimization protocols and their impact in kidney transplant fibrosis emphasizing the benefit to use calcineurin inhibitors, in particular tacrolimus. Takiyama et al. proposed hypoxia and hypoxia inducible factors (HIFs) involvements in diabetic kidney and discussed their pathophysiological relevance. In this review they summarized the diverse roles of HIFs from the several aspects in diabetic nephropathy well including kidney protective roles of antidiabetic drug metformin and sirtuins.

Kidney fibrosis is important research topic for both clinicians and research scientists. Today we have neither magic drugs nor miracle method to cure kidney fibrosis. Despite such limitation in real world, as shown in this special issue, recent advance in the kidney fibrosis research would provide some clues for combating kidney fibrosis. We hope this special issue provides sufficient and useful information for clinical/basic science researchers to design the therapeutic approach and the future research directions.

*Keizo  Kanasaki* *Akito  Maeshima* *Gangadhar  Taduri* *Ignacio  Revuelta*

